# Femtosecond Optoinjection of Intact Tobacco BY-2 Cells Using a Reconfigurable Photoporation Platform

**DOI:** 10.1371/journal.pone.0079235

**Published:** 2013-11-14

**Authors:** Claire A. Mitchell, Stefan Kalies, Tomás Cizmár, Alexander Heisterkamp, Lesley Torrance, Alison G. Roberts, Frank J. Gunn-Moore, Kishan Dholakia

**Affiliations:** 1 School of Physics and Astronomy, University of St. Andrews, St. Andrews, Fife, United Kingdom; 2 Biomedical Optics Department, Laser Zentrum Hannover e.V., Hannover, Germany; 3 School of Medicine, University of St. Andrews, St. Andrews, Fife, United Kingdom; 4 Institute of Applied Optics, Friedrich-Schiller-University Jena, Jena, Germany; 5 Cell and Molecular Sciences, James Hutton Institute, Invergowrie, Dundee, United Kingdom; 6 School of Biology, University of St. Andrews, St. Andrews, Fife, United Kingdom; University of Zurich, Switzerland

## Abstract

A tightly-focused ultrashort pulsed laser beam incident upon a cell membrane has previously been shown to transiently increase cell membrane permeability while maintaining the viability of the cell, a technique known as photoporation. This permeability can be used to aid the passage of membrane-impermeable biologically-relevant substances such as dyes, proteins and nucleic acids into the cell. Ultrashort-pulsed lasers have proven to be indispensable for photoporating mammalian cells but they have rarely been applied to plant cells due to their larger sizes and rigid and thick cell walls, which significantly hinders the intracellular delivery of exogenous substances. Here we demonstrate and quantify femtosecond optical injection of membrane impermeable dyes into intact BY-2 tobacco plant cells growing in culture, investigating both optical and biological parameters. Specifically, we show that the long axial extent of a propagation invariant (“diffraction-free”) Bessel beam, which relaxes the requirements for tight focusing on the cell membrane, outperforms a standard Gaussian photoporation beam, achieving up to 70% optoinjection efficiency. Studies on the osmotic effects of culture media show that a hypertonic extracellular medium was found to be necessary to reduce turgor pressure and facilitate molecular entry into the cells.

## Introduction

The delivery of functional molecules into living eukaryotic cells is a common research technique to study an organism’s physiology. Desirable compounds for introduction into cells can include nucleic acids for gene function and protein expression studies; biosensors for monitoring response to stimuli; as well as proteins, antibodies, dyes and drugs. However, the lipid bilayer of the cell membrane acts as a barrier to defend the cell against foreign molecules. A number of transfection techniques were developed to breach this barrier and deliver various molecules of interest into cells.

Crossing the cell membrane is considerably more challenging in plant cells compared to mammalian cells due to the additional presence of a cell wall. The cell wall can be up to 0.2 µm thick, 20 times thicker than the adjacent cell membrane (7–9 nm), and is selectively permeable to molecules smaller than approximately 4 nm in diameter [1]. Furthermore, the cell wall causes other complications, for example, during normal homeostasis, the cell membrane pushes against the wall, conferring an internal turgor pressure to the cell. This pressure may be increased if cells are bathed in a hypotonic culture medium [2] making it difficult to introduce molecules to the protoplast.

Current methods for molecule delivery into plant cells include microinjection [3]–[5], particle bombardment [6] and the application of cell-penetrating peptide (CPPs) [7]. Microinjection is a highly selective process but it requires skilled operators and very few injections can be achieved in a given time. Particle bombardment and CPPs can target large numbers of cells at once to achieve a higher frequency of delivery but suffer from a lack of specificity and in the case of bombardment, cell damage and death impacts transfection efficiencies. To overcome these limitations, the use of a tightly focused laser beam to increase the permeability of the cell membrane could provide a selective and minimally-invasive method for molecule deliver but with increased cell throughput compared to microinjection [8].

When compared to the rapidly-expanding mammalian cell photoporation literature [9], laser-mediated injection of molecules has rarely been used in plant cells. The first plant optical injection was demonstrated in [10] where a 343 nm nanosecond (ns) laser was used for the introduction of fluorescently-labeled DNA into *Brassica napus* cells without stating the efficiency of optoinjection. Other methods have also used a short-wavelength ns laser for plant cell photoporation [11]–[15] where cell permeability was achieved via heating or thermo-mechanical stress [16]. Awazu *et al* used an infrared (IR) ns laser beam to inject the nuclear-staining dye DAPI, and also DNA, into tobacco BY-2 cells but here it was reported to have a very low DAPI optoinjection efficiency (∼1–3%) [17].

Previously, femtosecond (fs) near-infrared (NIR) pulsed lasers have generally been found to be the most effective for single mammalian cell photoporation with inherent advantages over other laser-based systems [18]. The laser wavelength allows for deep penetration while the high repetition rate ultrashort pulses induce multiphoton absorption leading to photochemical effects in a limited focal volume. This approach minimizes any collateral damage to the cell structure [16]. Fs optical injection and transfection has proven to be valuable for many different mammalian cell lines, particularly hard-to-transfect cell lines such as neurons [19], [20], stem cells [21] and *in vivo* systems [22]. With regard to plant cells, high-precision fs laser-mediated optoinjection of single cells within Arabidopsis root was reported first by Tirlapur and König [23] and has been investigated further in Arabidopsis epidermal cells [24]. While it is useful to explore single-cell photoporation in higher plant tissue, this cannot be considered particularly representative of the plant cell system due to the individual cells’ specialized states. The tobacco BY-2 cells in suspension used in this study are chosen for their homogeneity and predictability, which makes them a widely accepted representative model system [25]–[27].

**Figure 1:**
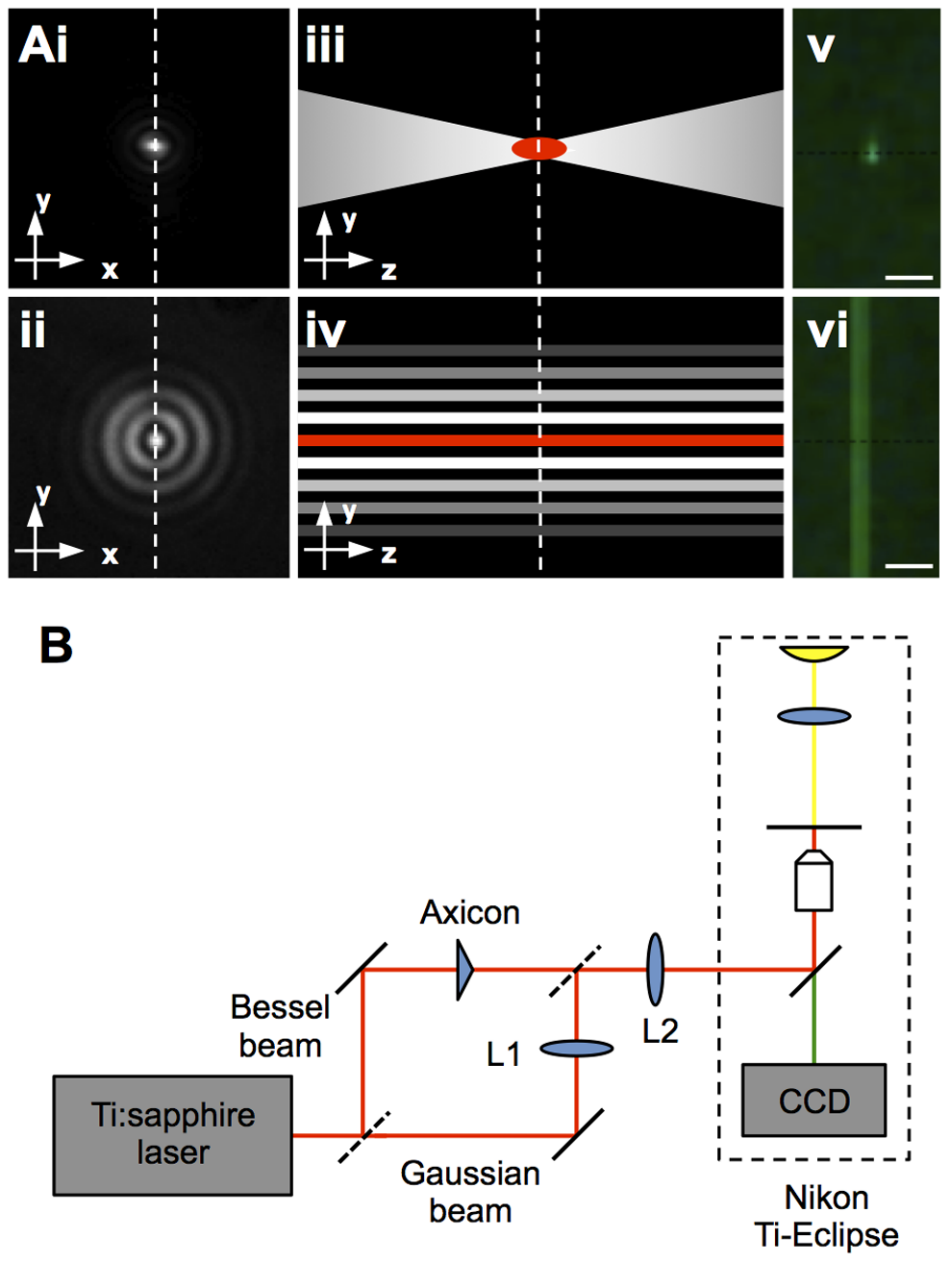
Optical set-up applied for plant cell photoporation. A provides a series of schematics depicting Gaussian and Bessel beams. The first column shows example microscope images of transverse profiles of focused Gaussian (i) and Bessel (ii) beams. Illustrative axial profiles are seen in iii and iv where the regions of multi-photon absorption are shown in red. The Bessel beam axial extent of multi-photon absorption is much longer than that of the Gaussian. The white dotted lines show the corresponding cross-sections of the orthogonal images. The final column experimentally shows two-photon excitation of fluorescein of each beam adapted from [30], scale bars represent 100 µm. Laser setup is shown in B. Output from the laser was directed into either of two arms using a removable mirror, denoted by dotted lines. A Bessel beam was generated using an axicon and a Gaussian beam spot was created using a system of telescopes (L1 and L2). The beam paths were directed into the back of a commercial inverted microscope where all imaging was performed.

In the majority of studies in this field, it was necessary for the laser to be tightly focused precisely at a specific point on the cell membrane. However, Tsampoula *et al* [28] demonstrated that for mammalian cells, fs optical transfection could be achieved over a wider range of axial distances from the cell membrane by using a Bessel beam [29]. This optical field has a transverse profile that is propagation invariant over a finite distance. Consequently it has a central maximum which extends several Rayleigh ranges compared to a standard Gaussian beam [30] (Figure 1). A Bessel beam is created when a Gaussian beam is incident upon a conical lens, known as an axicon. The resultant transverse intensity pattern consists of a central core surrounded by concentric rings [31] that can be described by a Bessel function of order zero. Interference effects within the Bessel beam lead to an increased axial propagation when compared to a focused Gaussian beam [32]. A longer axial propagation distance provides a large operating region over which the necessary multiphoton effects can occur, thereby essentially relaxing the requirement for tight focusing of the laser spot exactly on the membrane [30]. A more power-efficient membrane targeting method used a spatial light modulator (SLM) to sequentially apply axially separated Gaussian laser doses [33] and saw a 60% increase in optoinjection efficiency over a single dose in mammalian cells. This paper aims to directly compare Gaussian and Bessel light modes particularly to determine their effectiveness in a cell line in which the membrane is significantly more challenging to target when compared to the standard CHO-K1 model cell line. A third method to reduce membrane targeting eschews targeting single cells with a tightly-focused beam in favour of using weakly focused lasers to irradiate nanoparticles attached to cells in a large field of view [34]–[37]. The resulting plasmonic effects cause an increase in membrane permeability in a large number of cells in a short period of time, at the cost of single-cell selectivity.

As mentioned above, plant cells are reliant on turgor pressure to maintain their shape. Standard plant culture medium is hypotonic with respect to the cell interior so the creation of a pore in the membrane initiates an outward flux of cytosol as the osmotic pressure is equalized, seen in Guo *et al* [12]. Hypertonic treatment of the cell causes plasmolysis, creating a “temporary protoplast” as the membrane is pulled away from the cell wall and laser access to the cell membrane is enhanced [38]. A breach of the cell membrane will then cause uptake of extracellular medium by the cell. Ferrando *et al* showed that subjecting plant cells to high osmolarities during plasmolysis-deplasmolysis cycles can cause high cell death rates [39]. Guo *et al* reported a transformation efficiency of only 0.5%, but the reasons for the low transformation efficiency were not elucidated and could be due to frequent cell death induced by the large osmotic change in medium applied for the poration of cells in this study [12]. Media which are only weakly hypertonic, however, reduce the void area produced [39] and therefore decrease the maximum possible medium uptake by the plasmolyzed cells. By incrementally changing osmolarity and studying the effects on both cell death and medium uptake, it would be possible to optimize photoporation in plant cells.

In this study, the optimal parameters that determine fs optical injection of intact plant cells are evaluated and described. An experimental setup, allowing reconfiguration between a Gaussian and a Bessel beam at the focus by switching optical components, was used to vary optical parameters. Comparisons between the two optical geometries were conducted on tobacco cells in culture (*Nicotiana tabacum* L., cv Bright Yellow 2 (BY-2), a model plant cell line [40]) to determine the effect of beam geometry on plant cell optoinjection and subsequent viability. In addition, cells were photoporated in media of differing osmolarity and the effect on optoinjection efficiency and cellular uptake of membrane impermeable molecules was monitored.

## Results

### Bubble Formation and Optoinjection Success

**Figure 2:**
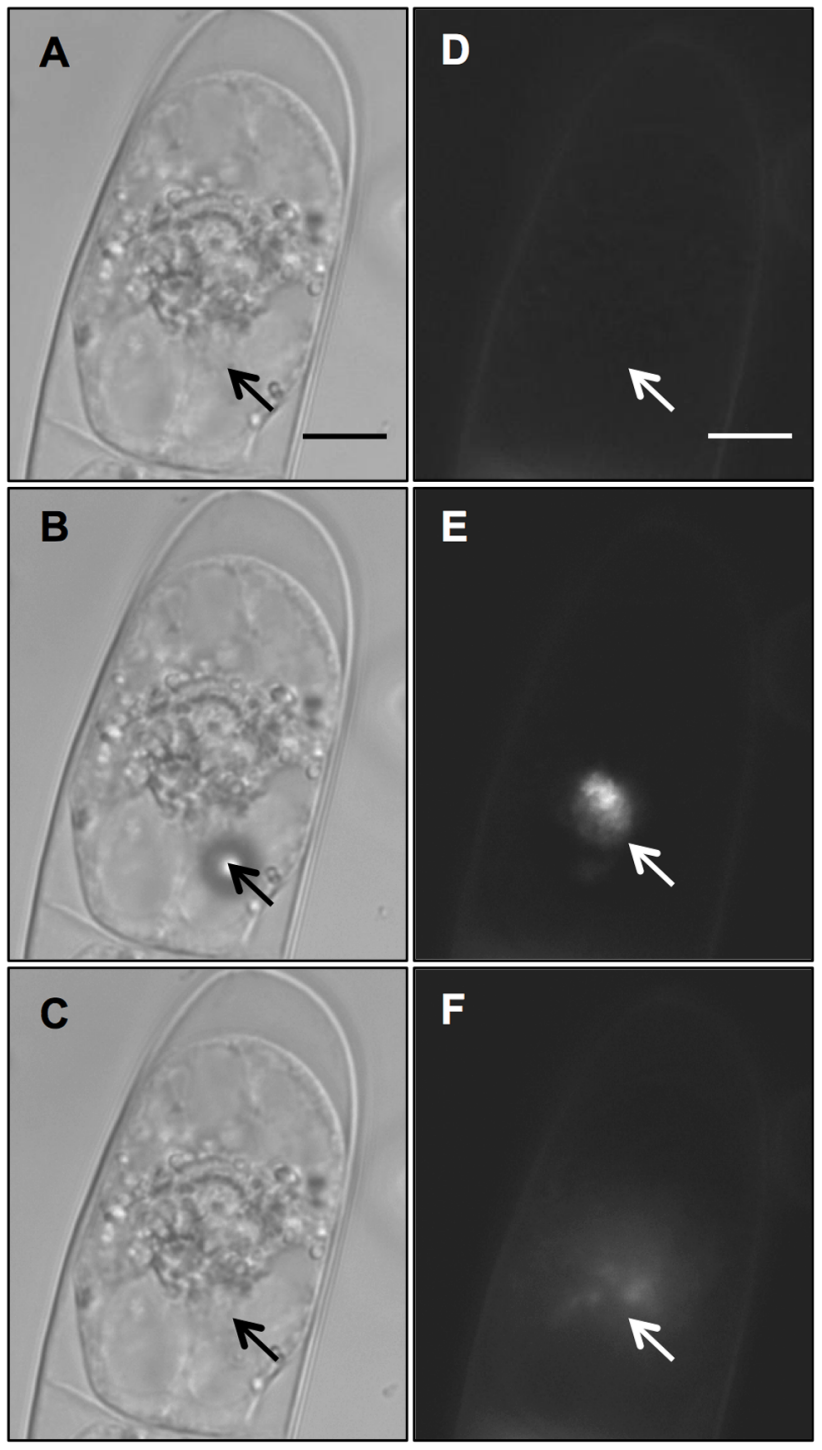
Optical injection of PI into a BY-2 cell. Shown in bright field (A–C) and fluorescence (D–F). A) Before shooting, B) transient bubble created on cell membrane during laser dose, C) no visible laser damage left post-irradiation. D) Pre-irradiation showing faint PI staining of the cell wall. E) Laser induced transient auto-fluorescence at the point of irradiation. F) Permanent increase in cytosolic fluorescence as PI enters the cell. Arrows indicate site of laser irradiation. Scale bar denotes 10 µm.

Successful optoinjection by both beam geometries of the BY-2 cells always was preceded by the creation of a gas bubble at the cell surface as seen in Figure 2B. These bubbles are caused by multi-photon absorption leading to photo-ionisation within the focal volume. If the density of free electrons created exceeds the optical breakdown threshold of the irradiated material then a cavitation bubble will be produced [18], disturbing the cell membrane and transiently increasing its permeability. By increasing the laser fluence, long-lasting gas bubbles, of the order of a few seconds, with larger diameters (≥5 µm) could be created; however, these forms of bubbles were observed to be followed by cell death as damage to the membrane is permanent [41]. The transient nature of the bubble at low laser intensities is shown in Figure 2C, which was taken 3 minutes after a laser dose and shows no lasting visible damage to the area of irradiation.

### Determining the Effect of Changing Optical Parameters on Optical Injection

The effect of the beam geometry on successful photoporation was determined by irradiating the cells with either a Gaussian beam or a Bessel beam. The initial experimental conditions used standard culture medium made hypertonic by the addition of 0.29 M sucrose as the surrounding medium to induce plasmolysis of cells. Sucrose was chosen as an osmoticum due to the high viability it allows during plasmolysis-deplasmolysis cycles when compared to inorganic solutes [42].

The diameter of the central spot of the Bessel beam (2r_0_) was matched to the beam waist of the focused Gaussian beam (2ω_0_) and the axial extent was approximately 13 times longer than the Rayleigh range of the Gaussian beam. This is the axial length in which all multi-photon events occur (see text S1). Two different modes of laser irradiation were employed using the Gaussian beam; either a single dose or three doses separated by approximately 2 µm axially and 1 second temporally, the latter is intended to increase the chance of targeting the cell membrane while avoiding any accumulative effect from multiple exposures [33]. A single shot was applied with the Bessel beam. In this way we could compare the two methods previously utilised to increase the chance of targeting the cell membrane alongside the standard single Gaussian dose. The effect of the laser intensity applied to the cell was investigated as it has previously been shown that the laser fluence affects the efficiency of optoinjection [43].

PI was chosen as the optoinjection fluorophore for this part of the experiment since the lack of background fluorescence makes small uptake volumes easy to image. PI is membrane impermeable unless the cell membrane is compromised and it is used as a standard proof-of-concept photoporation fluorophore [44]–[46]. Upon entry into a photoporated cell, PI intercalates with nucleic acids present in the cytosol causing enhanced fluorescence [24], which is seen experimentally in the cytoplasm. Optoinjection of PI into the cell can be seen in the fluorescent images from Figure 2. Prior to laser irradiation, no background fluorescence is seen except a weak staining of the plant cell wall (Figure 2D) caused by PI binding to pectins in the cell wall [47]. Upon irradiation, a broadband autofluorescence was induced at the laser focus, as shown in Figure 2E. This effect was either transient or permanent depending upon laser intensity, with permanent autofluorescence indicating cell death. If photoporation was successful, entry of PI into cells occurred and cytosolic fluorescence was observed (Figure 2F).

**Figure 3:**
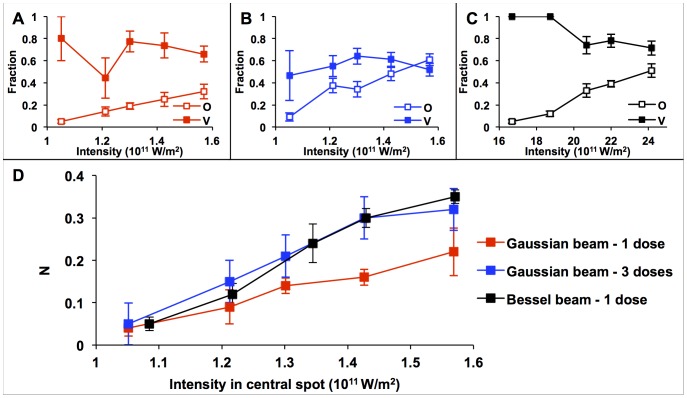
Optoinjection efficiency (O) and viability (V) of the BY-2 plant cells at different laser powers. After irradiation by (A) three shots or (B) a single shot with the Gaussian beam or (C) a single shot with the Bessel beam. For both beam geometries the optoinjection efficiency (represented by open squares) increases with power at the focal plane while viability (solid squares) usually decreases. C shows N (the proportion of cells being both viable and optoinjected) for varying central spot intensities. N increases as the intensity increases. The Bessel beam (black) shows a higher value for N than the Gaussian beam (red) when considering a single shot. When comparing with three axially separated shots of the Gaussian beam (blue), N is comparable to the Bessel beam. Each data point represents the mean for n = 5 with 20 cells per experiment. Error bars represent the standard error of the mean (S.E.M.).

The application of each of the three laser irradiation patterns displayed increasing efficiency of optoinjection (O) as the intensity within the central spot (where all multi-photon effects are assumed to occur) increased (Figure 3A,B,C). Three doses with the Gaussian beam (3B) displayed the highest efficiency, achieving up to 61±5%, although viability was severely compromised in this regime, never rising above 65%. Single shots with the Gaussian beam (3A) showed the lowest efficiencies overall, (maximum efficiency of 32±7%) but with greater viability than with three doses. The Bessel beam (3C) displayed the highest viabilities at the majority of intensities and provided intermediate efficiencies, reaching a maximum at 51±6%.

In order to compare quantitatively the beam geometries, we consider which irradiation pattern would produce the highest proportion of cells that are both optoinjected and viable (defined as N). To determine N, the product of the optical injection efficiency (O) and viability (V) was calculated at each central beam spot intensity (Figure 3D). The percentage of the power in the central spot of the Bessel beam was calculated to be 6.5%. As the area of the central spot is smaller than the surrounding concentric rings, only the central spot has a high enough intensity to create multi-photon interactions and therefore contribute to photoporation [16]. At low laser intensities, N is small due to the low optoinjection efficiency even though the viability of the optoinjected cells can be up to 100%. As the intensity of the laser increases, so does N as O begins to increase with only a slight reduction in viability. Figure 3D shows that at higher intensities, the single shot Gaussian provides the lowest values of N. For all intensities explored in this study, the Bessel beam and three shots with the Gaussian beam display comparable values for N; the higher efficiencies achieved when using the Gaussian beam are counteracted by the subsequent decrease in viability.

### Effect of Medium Osmolarity on Optoinjection Efficiency

Having established that the Bessel Beam geometry was the optimal configuration for time-efficient optoinjection of dyes into BY2 tobacco plant cells, the potential role of the extracellular medium on optoinjection efficiency was investigated. Prior to laser irradiation in the presence of PI, cells were incubated in media using differing sucrose concentrations to vary the osmolarity, starting with the standard hypotonic medium used for culturing (total osmolarity of 171±2 mOsm/L). Experiments were performed using a single 40 ms dose from the Bessel beam with a power of 1.6 W at the focal plane to maximise photoporation efficiency.

**Figure 4:**
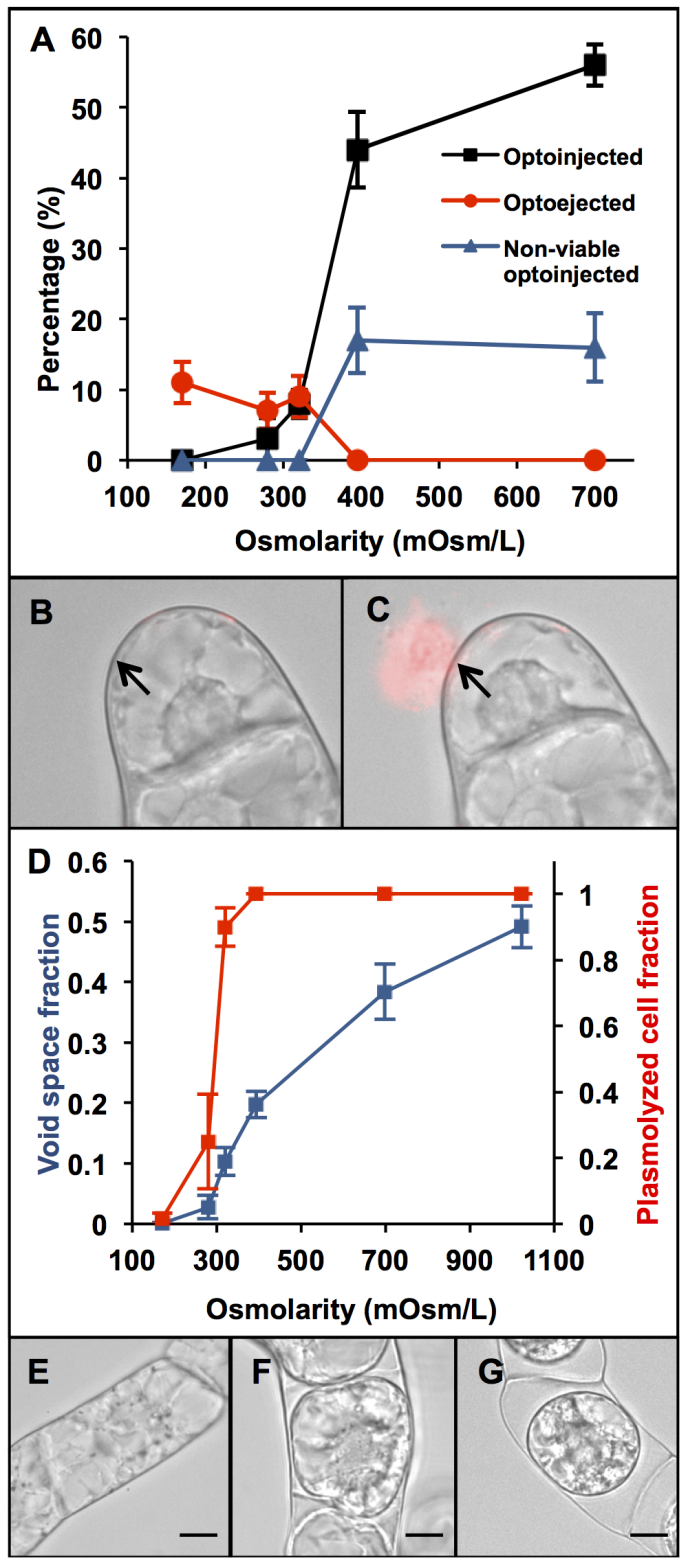
Optoinjection efficiency differs depending upon the osmolarity of the surrounding medium. From looking at A, as we increase the osmolarity, the efficiency of optoinjection (black squares) increases from zero up to 50% as the surrounding medium changes from hypertonic to hypotonic. Conversely, the optoejection efficiency (red circles) falls from around 10% to 0% above 320±2 mOsm/L. As the molarity of the solution increases, the likelihood of cell death (represented by blue triangles) is increased. At 320±2 mOsm/L the optoinjection and ejection efficiency are approximately equal. Each data point was performed in triplicate with 20 cells; error bars represent S.E.M. B and C show brightfield and fluorescence overlays of a cell prior to (B) and 2 minutes after (C) laser irradiation in standard culture medium in the presence of PI. Photoporation induces a flux of cytosol from the cell into the extracellular medium. The cytosol ejected from the cell becomes stained with PI and a bright fluorescence is seen exterior to the cell. Large changes in cell morphology occur and cell death is induced. D shows the osmotic effect on plasmolysis of BY-2 cells. Both the void space (blue) and fraction of plasmolyzed cells (red) increases with the osmolarity of the surrounding medium but the red line shows a much steeper incline around the point of incipient plasmolysis (50%). Error bars denote the S.E.M. for n = 3 experiments with 20 cells counted in each. E-G show example cells in standard culure medium (171±2 mOsm/L), very weakly hypotonic (320±2 mOsm/L) and strongly hypotonic (699±4 mOsm/L) solutions respectively, with the resulting plasmolysis occurring slightly in F but seen very strongly in G as the membrane pulls away from the cell wall in the highly osmotic solution. Scale bars denote 10 µm.

At each of the five osmolarities tested, cells were photoporated and studied for either injection or ejection of cytosol from the protoplast (Fig. 4A). Cells were also screened for viability as described in the previous section. At low osmolarity (lower than 320±2 mOsm/L) the primary effect observed was an ejection of cytosolic medium into the extracellular environment after laser treatment (Figure 4A,B). Extracellular PI then binds to solutes ejected from the cell to cause an increase in fluorescence around the exterior of the cell (Fig. 4B). Conversely, at osmolarities greater than 320±2 mOsm/L, the dominant effect was an intake of extracellular medium (injection, Fig. 4A). In contrast to optoejection, optoinjection was characterized by fluorescence within the protoplast (Fig. 2F). The increase in injection frequency, however, was counteracted by an increase in the occurrence of cell death in optoinjected cells. Figure 4A shows that in media with an osmolarity of 320±2 mOsm/L, optoinjection and optoejection both occur in about 10% of cells.

To relate these photoporation effects seen to plasmolysis changes within the cell, the degree of plasmolysis at the different solution osmolarities was measured by the two methods usually employed [48]: measuring the void space and counting the number of plasmolyzed cells. By looking at the void space as a fraction of the whole cell (Figure 4D), a general increasing trend is observed as the osmolarity of the surrounding solution is increased. This trend begins slowly at 171–279 mOsm/L, is steepest around 279–395 mOsm/L and then starts to level off again after 395 mOsm/L. The number of plasmolyzed cells seen in each solution also shows an increase with osmolarity, but with a much sharper growth between 171–320 mOsm/L followed by a saturation point at 399 mOsm/L, beyond which all of the observed cells were plasmolyzed. Examples of cells in low, medium and highly osmotic solutions are shown in Figures 4E–G respectively.

### Exploration of Optoinjection Dynamics under Hypertonic Treatment

Next we explored the dynamics of optoinjection in more detail. Calcein was used to monitor the continuous uptake of extracellular medium during photoporation of intact cells in media of differing hypertonic osmolarities (395±6, 699±4 and 1024±11 mOsm/L; abbreviated to 0.4, 0.7 and 1 Osm/L in the following section). These measurements allowed us to establish the time-scales over which optoinjection occurs and also the volume of extracellular medium that is taken up and how these change with extracellular osmolarity. The small size and inert nature of calcein makes it an ideal fluorophore for monitoring cellular uptake, moreover there is no time delay (as occurs with PI) in visibility of the fluorescence inside the cell. As described above, three axially separated shots using the Gaussian beam at 70 mW at the focal plane were used for optoinjection, taking advantage of the power-efficiency of the Gaussian beam. The increase in intracellular fluorescence was observed using a confocal microscope to eliminate background fluorescence.

For each solution osmolarity a least squares fit was performed in order to determine the asymptotic value for the maximum fluorescence (the value reached if we could observe at later timescales, given by I_max_). This strategy avoids selection of spurious maxima and gives an accurate estimate of the late time behaviour. The asymptotic value of the maximum fluorescence reached has a direct correspondence to the volume of extracellular medium taken up by the cell as a fraction of the cell volume. Using these fitted curves also allows us to establish the time taken to reach 50% of that value (t_1/2_), which gives an indication of the rate of medium uptake.

**Figure 5:**
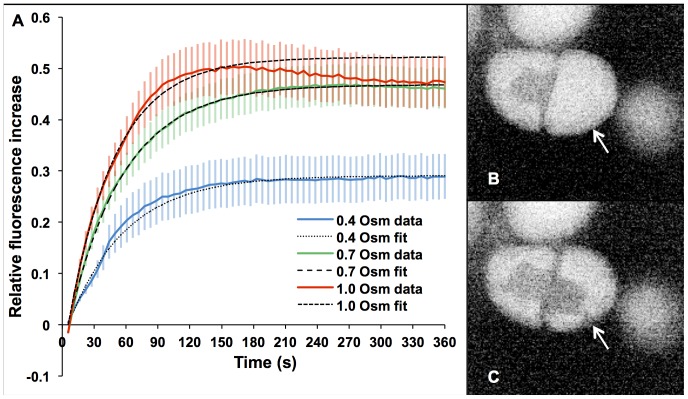
Uptake of calcein during photoporation in hypertonic medium. Solid lines in A show the mean increase in intracellular fluorescence relative to background for n = 20 cells with error bars representing 0.5 S.E.M. for clarity. Each curve shows a sharp increase in fluorescence in the first few minutes after photoporation, which plateaus after around 3 minutes. The higher molarity solutions show a quicker increase in calcein uptake and reach a higher level of maximum fluorescence than the lower molarity solutions. Dashed lines denote fitted saturation curves. B and C show a cell (arrowed), in negative contrast for clarity, before and 60 seconds after photoporation in 0.4 Osm/L medium containing calcein. The nucleus, indistinguishable from the rest of the unporated cell in B, becomes filled with calcein along with a cytosolic strand (just visible at arrow tip) but none enters the large vacuole surrounding it.

It can be seen from Fig. 5A that for each solution, the average fluorescence increases relative to the background and shows a sharp increase in the first few minutes followed by a trend towards a horizontal asymptote. The 1.0 Osm/L curve decreases once a maximum point is reached which could indicate the onset of cell death caused by the large physiological changes discussed in the previous section. Data for the first 200 seconds only are used to perform the fit for the 1.0 Osm/L curve and thereafter extrapolated to accurately fit the initial rapid increase. This allows more appropriate values for I_max_ and t_1/2_ to be determined which are independent of the observed decrease.

In Table 1, the asymptotic maximum fluorescence for the different molarity solutions is compared. As the molarity increases, I_max_ also increases, more than doubling between 0.4 and 1.0 Osm/L. From inspection of the time taken to reach half of the maximum fluorescence (t_1/2_, Table 1), we can gain an insight into the rate at which calcein is taken up by the cell. Each of the curves appears close to saturation after three minutes (Figure 5A) but Table 1 shows that higher molarity solutions induce a greater uptake rate, although the change is less pronounced than for I_max_, with a decrease of less than 20% occurring between 0.4 and 1.0 Osm/L.

**Table 1:** Parameters determined from non-linear fits of calcein uptake.

Medium Osmolarity (Osm/L)	I_max_	t_1/2_ (s)
0.4	0.291(7)	37(1)
0.7	0.468(3)	36.3(3)
1.0	0.521(9)	30.8(5)

As the osmolarity increases, the asymptotic maximum fluorescence value, which the saturation curve tends towards, increases. The time taken to reach 50% of the maximum fluorescence decreases as the molarity of the solution increases. The increased pressure difference caused by higher molarity solutions induces more and quicker uptake of medium to balance it. Brackets denote the error in the final digit; uncertainties were calculated from the R-squared value of the fitted curves.

An example of calcein optoinjection before and after photoporation is shown in Figures 5B and C. The confocal image is shown in negative to better highlight the entry of the calcein (dark) into the cell (light). Prior to photoporation, the protoplast interior is free of calcein and surrounded by strong background fluorescence. After photoporation, the nucleus and a thin cytosolic strand can be seen to be filled with calcein as they turn dark.

### Effect of Fluorophore Size on Cellular Uptake

Although fluorophores with a molecular weight of less than 1 kDa are useful for investigating and optimizing the optical injection process, biologically-relevant compounds are usually much larger than this, with proteins and DNA reaching up to hundreds of kDa in size. The presence of the cell wall might therefore present a problem in the delivery of these molecules. To investigate this, fluorescently-labeled dextrans of varying sizes were optoinjected into both intact BY-2 cells and BY-2 protoplasts. Dextrans are non-ionic polysaccharides available in specific weights that are frequently used in membrane exclusion studies. Cells were optoinjected using the same irradiation parameters as the preceding experiment. Confocal images were taken prior to irradiation and 3 minutes afterwards to monitor how cellular uptake changes with optoinjectant size.

**Figure 6:**
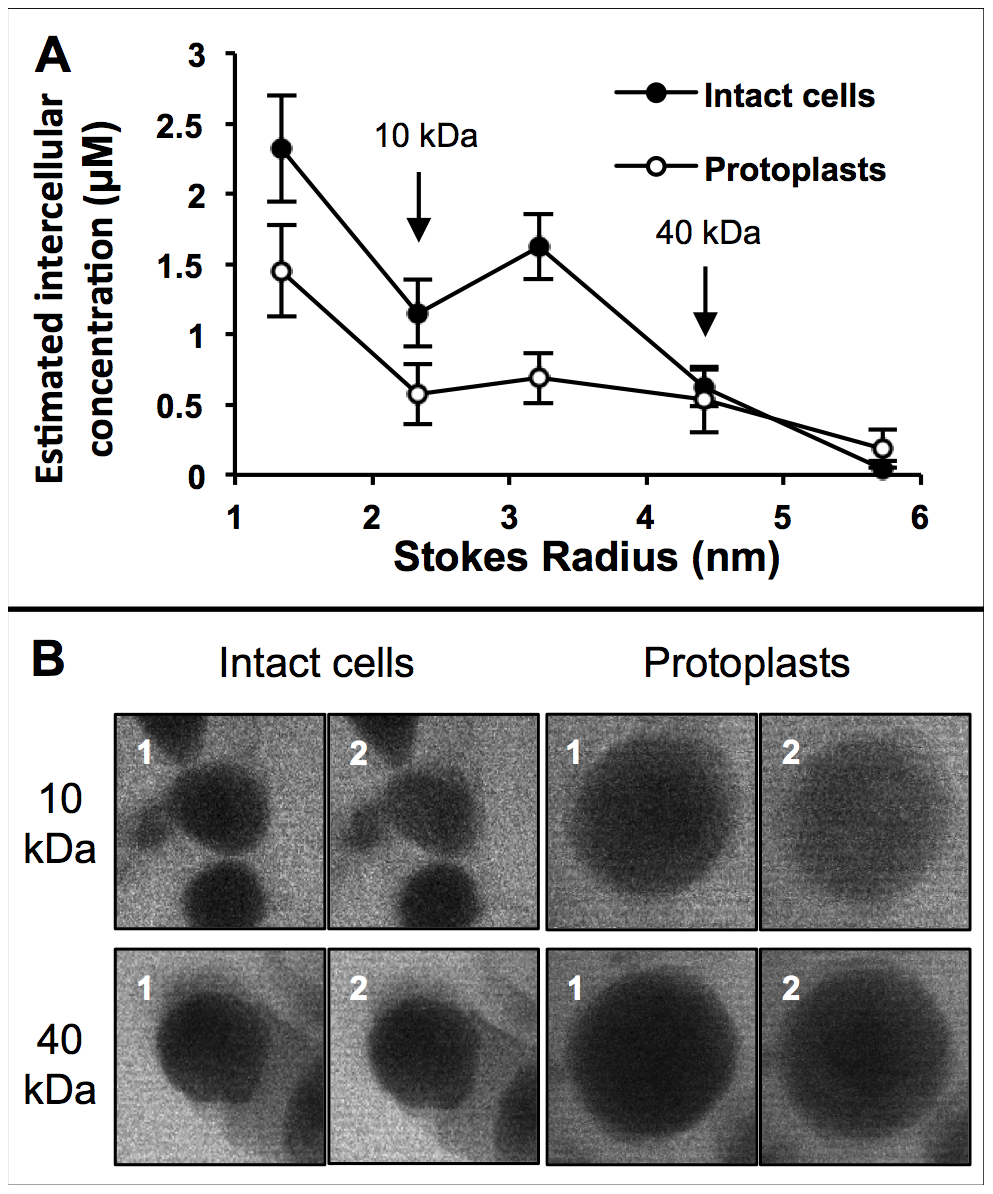
Effect of molecule size on cellular uptake. From looking at A, it can be seen that as the Stokes radius increases, the amount of dextran taken up by the cell decreases. The protoplasts take up less dextrans than the intact cells for Stokes radii smaller than 5 = 30 cells with error bars representing the S.E.M. B shows representative images (those depicting uptake most comparable to the average uptake) of intact cells and protoplasts before (1) and 3 mins after (2) photoporation in the presence of small and large dextrans. The larger dextrans show less (though still visible) entry into the cell than the smaller dextrans.

The effect of dextran size on cell uptake can be seen in Figure 6. The Stokes radius (SR) is calculated from the molecular weight (MW) using the empirical formula SR[nm] = 0.81*(MW[kDa])^0.46^, taken from [49], [50]. Both intact cells and protoplasts display decreased cellular uptake as the Stokes radius increases. For Stokes radii equal to and smaller than 4.42 nm (corresponding to 40 kDa dextrans), the protoplasts show less cellular uptake than the intact cells. At 3.2 nm (20 kDa), the cellular uptake for intact cells is three times greater than for protoplasts. At 5.71 nm (70 kDa), the cellular uptake is severely reduced in both cell types, although four times more dextrans are taken up by the protoplasts than the intact cells.

## Discussion

This paper demonstrates the optical injection of various fluorophores into intact plant suspension cells using an ultrashort-pulsed laser. It was found that by varying the optical and biological parameters, the optoinjection efficiency and dynamics vary greatly.

By increasing laser intensity, the optoinjection efficiency was maximized for both beams because a larger disruption to the cell membrane occurred. The more severe the disruption to the membrane, the more likely that the cell will be non-viable post-irradiation as the membrane permeability may become permanently compromised. The maximum optoinjection efficiency achieved with either beam was over 10-fold higher than those presented previously applying a ns laser to intact BY-2 cells [17] and similar to those achieved using a femtosecond Gaussian beam in Arabidopsis epidermal cells [24].

When considering the application of a single dose, using the Bessel beam over the Gaussian beam provided a higher efficiency of optoinjection, producing nearly 50% more viable optoinjected cells, shown in Figure 3D. This could lead to an increase in throughput in plant cell optoinjection as the total number of cells that require targeting to achieve a certain number of usable cells is reduced. The most time-consuming part of the optoinjection process is aligning the membrane with the focal plane of the focused Gaussian beam. With the plant cells this is made even more challenging as (along with the difficulty of the cell wall) plant cells are much greater in size, barrel-shaped, non-adherent and relatively less homogeneous in morphology than mammalian cells. The long propagation invariance of the Bessel beam makes it easier to target the membrane without the necessity for precise focusing beforehand, reflected in the higher optoinjection efficiencies achieved. The time spent focusing is then reduced and the number of cells that can be photoporated in a given time is increased. Figure 3D also showed that it is possible to increase the efficiency of Gaussian beam photoporation to match that of the Bessel beam by using multiple doses. While this is useful in systems where power efficiency is critical, it again reduces the possible cell throughput due the extra time required to apply the doses and manually align the stage above and below the focal point. Using the Bessel beam, approximately 300 cells could be irradiated per hour which is three times higher than an automated protoplast microinjection system [51]. This frequency could be further increased with the inclusion of automated cell targeting [52], raster-scanning system [33] or microfluidic technology [41], [53].

The long axial propagation of the Bessel beam and the self-healing properties may also prove useful when considering other plant cell types. While the BY-2 cells are genetically identical to their parent plant *Nicotiana tabacum* and it can be assumed that the cell wall will be of a similar composition, differing plant species and cell types will portray slightly differing cell wall properties, although the primary constituents of cellulose and hemi-cellulose will remain. If other cell types requiring optoinjection displayed thicker secondary cell walls then the Bessel beam could help to bypass this issue, though a higher power may be required in order to counteract any abberations introduced to the beam by passing through this cell wall. Another option to bypass a thicker cell wall could be to employ wavefront shaping [54].

Changing the osmolarity of the solution the cells were bathed in allowed us to affect the delivery of molecules into the cell. At low osmolarities, the cell is fully turgid and the cell membrane is pushed against the cell wall by osmotic pressure (Figure 4E). Therefore breaching the membrane allows cytosol to flow from the cell, reducing the pressure within the cell and ultimately rendering it non-viable. At higher osmolarities the opposite effect occurs, with the difference in pressure caused by the osmotic gradient that draws material into the cell. Higher osmolarities, however, also caused increased cell death as the amplitude of physical changes induced in the cell was increased. Higher osmolarity media induce larger voids between the cell membrane and cell wall (Figure 4G), which are then partially refilled as medium enters the cell upon photoporation. Low cell viability following photoporation in high osmolarity media has also been seen in mammalian cells [55].

The crossover between optoejection and injection occurring at 320±2 mOsm/L could represent the approximate internal molarity of the average BY-2 cell. Figure 4D shows the point of incipient plasmolysis (taken to be where 50% of cells are plasmolyzed [56]) occurs between 279 and 320 mOsm/L. Incipient plasmolysis is the point at which the cell membrane just starts to pull away from the cell wall, the surrounding medium is therefore deduced to be isotonic. This is also supported by looking at the void fraction which increases quickest around 279 and 395 mOsm/L. It appears that the fraction of cells plasmolyzed is a more sensitive measure of the cell osmolarity whereas determining the void space better represents the plasmolysis effects at higher osmolarities, where the first method saturates. The primary mechanism of molecule delivery at an isotonic osmolarity will be *via* diffusion, akin to mammalian cell optical injection [33].

Complementary effects were seen when temporally monitoring cell uptake for differing osmolarities. The increased I_max_ at higher osmolarites is due to the larger osmolarity gradient between the intra- and extracellular medium producing a greater plasmolyzing effect which creates a large pressure differential across the cell membrane. This is also supported by the larger void space at higher osmolarities, as measured in Figure 4D. A greater volume of the highly osmotic solution must be taken up by the cell in order to balance the pressure, hence we see a greater uptake of calcein. The reduced t_1/2_ is also caused by the larger pressure differential induced by higher osmolarity solutions creating a higher inward flux of extracellular medium in accordance with mass conservation laws and has been observed on mammalian cells too [55].

This study also evaluated the effect of changing the fluorophore to be optoinjected. While it was observed that PI and calcein freely diffused into the cell upon photoporation, using dextrans of increasing MW adversely affected the amount of dextran that could enter the cell. Increasing the Stokes radius of the molecule decreases the diffusion coefficient, decreasing the likelihood of molecules entering the cell during the transient pore opening; this effect was seen in both intact cells and isolated protoplasts. Intact cells showed higher intracellular concentrations of dextrans at low MW than isolated protoplasts. This is most likely due to the osmotic pressure present in the plasmolyzed intact cells actively drawing more extracellular medium into the cell upon photoporation; isolated protoplasts rely solely on diffusion to optically inject the dextrans [57]. For higher MW dextrans the cell wall begins to affect the number of molecules that can be porated into the intact cell protoplast. Even before photoporation has taken place, as the size of the dextran added to the medium increases, the fluorescence within the apoplast decreases until at 70 kDa almost no fluorescence is seen in the apoplast, this will limit the dextran concentration next to the cell membrane and therefore the number of molecules which can be photoporated into the intact cell protoplast. Attempts to directly target the cell wall saw no increase in fluorescence in the apoplast, implying that photoporation only affects the cell membrane. The reduction in cellular fluorescence beyond 40 kDa suggests that the exclusion size of the cell wall for dextrans is between 4.42 and 5.71 nm (although partial exclusion occurs at lower Stokes radii) which supports previous experiments which put the dextran cell wall exclusion size at between 4.6 and 5.5 nm [58]. The isolated protoplast data suggest that it could be possible to use photoporation to inject molecules larger than 70 kDa past the plant cell membrane, although with very low intracellular concentrations achievable. For comparison, in animal embryonic cells dextrans of up to 500 kDa have been optoinjected [46].

## Conclusion

This study has demonstrated the potential for optical delivery of membrane impermeable molecules into intact plant suspension cells. When compared to the current molecule delivery methods, optoinjection can provide increased cell throughput (which reduces the time required to inject large numbers of cells) while still maintaining high efficiency and single-cell selectivity. The viability of successfully optoinjected cells was found to be high when compared to other molecule delivery techniques [59]. It was also demonstrated that the Bessel beam provides a more effective optoinjection method than with Gaussian irradiation, although the Gaussian beam is more power-efficient and simpler to implement.

External osmotic pressure was found to be critical to be able to inject cells with the compound of interest. Higher osmolarities show an increased efficiency of uptake and greater allowable volume of molecule. Increasing the osmolarity, however, also increases the amplitude of physiological changes within the cell and increases the chance of cell death occurring so a compromise needs to be made between these effects.

The size of molecule for delivery was found to be limited in intact cells to between 40 and 70 kDa. This limit may be representative only for polysaccharides and other similar molecules though because cell wall permeability has been shown to vary for differing molecule types e.g. globular proteins and ionic DNA [32]. It could also prove useful for future experiments to perform fluorescence correlation spectroscopy on solutions of dextrans to confirm that larger dextrans have more difficulty accessing the interior of the cell in both protoplasts and intact cells. For injecting larger molecules, isolated protoplasts might prove to be a more useful receiver vessel, although the number of molecules entering the protoplasts will still be low and protoplasts have limited use in research due to the difficulty in regeneration from them.

Possible future experiments could include using FRAP (fluorescence recovery after photobleaching) to investigate the membrane properties post-photoporation [60]. Potential applications for this technology include extensions to the injection of biosensors and other functional molecules or for transduction and transfection experiments. In addition the inherent properties of the Bessel beam may also allow access to deeper tissue.

## Materials and Methods

### Photoporation System

The output beam from a Ti:sapphire laser with a central wavelength of 800 nm, 140 fs pulse duration, 4 W max average power and 80 MHz pulse repetition rate (Chameleon Ultra II, Coherent Inc, USA.) was passed through a mechanical shutter (Newport, USA) capable of providing short exposure times on the order of tens of milliseconds. It was could then be redirected into either of two arms using a removable mirror. The photoporation experiments used powers of 0.07–0.120 W for the Gaussian beam and 1.2–1.8 W for the Bessel beam at the focal plane. The Gaussian beam was magnified using a 4x telescope and coupled directly into an inverted microscope (Ti-Eclipse, Nikon UK Limited, UK). An air objective (Nikon, 60x NA = 0.8) was used for optoinjection yielding a Gaussian beam waist diameter of 2ω_0_ = 1.0±0.1 µm with a corresponding Rayleigh range of 2z_R_ = 2.0±0.2 µm at the focal plane. An illuminated axicon (CVI Melles Griot, UK) of opening angle 5° created the Bessel beam that was magnified using a 4x telescope which, combined with the 15x demagnification provided by the tube lens and objective, gave a Bessel beam with a central core diameter of 2r_0_ = 1.0±0.2 µm and a propagation distance 26±2 µm at the focal plane. Figure 1A compares the intensity profiles of Gaussian and Bessel beams, including the regions of multi-photon absorption. Figure 1B shows a diagrammatic optical set-up of the system as described in this section.

The intensity of the laser in the central spot was determined by dividing the power within the central spot by the beam area. In the case of the Bessel beam, the power in the central spot was estimated using ImageJ [61].

### Culture of BY-2 Cells

BY-2 tobacco cells obtained from the James Hutton Institute were cultured according to Brandizzi et al [40] in Murashige and Skoog medium (MP Biomedicals, USA) supplemented with 1 mM 2,4-dichlorophenoxyacetic acid and 0.09 M sucrose in 20 ml conical flasks. During liquid cultivation the flasks were kept on an orbital shaker (IKA Labortechnik, Germany) at 120 rpm at 25°C. Cells were subcultured weekly at a 1 in 20 ratio.

### Photoporation of BY-2 Cells

Prior to the experiment, cells were collected by centrifuging 1 ml of a 3–5 day-old culture at 500 g for 2 min. The standard culture medium was aspirated and replaced with 500 µl of medium containing varying sucrose concentrations (from 0.09–0.69 M sucrose) depending on the experiment. The cells were then left at room temperature for 30 minutes to allow plasmolysis to occur. 100 µl of this solution was plated on a 10 mm glass-bottom dish (World Precision Instruments, USA) with an optical thickness of 0.17 mm. The cell membrane of individual cells was then targeted with 40 ms laser doses: either a single shot or three 2 µm axially separated shots (one shot focused on the membrane, the other two 2 µm above and 2 µm below the membrane). Axial separation was performed manually using a calibrated stage.

The osmolarity of each solution was measured using a freezing-point osmometer (Type 15, Löser, Germany) with each solution measurement performed in triplicate.

### Propidium Iodide Optical Injection

Just prior to laser irradiation, propidium iodide (PI) was added to a final concentration of 1.5 µM (Life Technologies, USA). The working concentration was empirically determined so that cell death was minimized after 1 hour in solution. PI was added post-incubation to reduce contact time with cells and therefore maximize cell viability.

Determination of optoinjection success was performed 2–3 minutes after photoporation using epi-fluorescence imaging with a cooled CCD camera (Clara, Andor, UK) and a TRITC filter cube (Nikon UK). Successful, viable optoinjection manifested as a low-level fluorescence over the protoplast interior. Extracellular fluorescence was indicative of optoejection having occurred. Unsuccessful photoporation would display either no fluorescence or only localized autofluorescence, which could be identified by scanning the imaging plane over the entire cell volume; autofluorescence was seen to only be present at the site of laser irradiation. Photoporated yet non-viable cells were identified by a strong PI fluorescence in the cell nucleus.

### Measurement of Plasmolysis

The degree of plasmolysis induced at different solution osmolarities was determined by two methods. Cells were incubated in osmotic solutions as described above. For each experiment, 20 cells were selected at random and the CCD was used to image a cross-section in brightfield. To measure the void space, both cell and protoplast were outlined manually in ImageJ to measure their areas. The number of cells that displayed plasmolysis for each experiment was also noted.

### Optoinjection of Calcein

The final working concentration of calcein (a membrane impermeable variant of the common viability fluorophore, calcein-AM) was 30 µM (Life Technologies), which was added just prior to irradiation. Calcein uptake was monitored using the 488 nm laser attached to a confocal imaging head (C1, Nikon UK); a cross-sectional image of the cell in the plane of laser irradiation was taken every 5 seconds for 6 minutes. Analysis of the images was performed using ImageJ [61]. The normalized fluorescence within the whole cell relative to the background was established.

Due to confocal imaging occurring during laser irradiation, it was impossible to screen the cells during irradiation for bubble formation (assumed to be the catalyst for optoinjection). Post-experiment determination of optoinjection success was performed by using a 3σ threshold on the fluorescent data: if the maximum fluorescence reached was greater than three times the standard deviation of a control cell in the same field of view then the cell was assumed to be optoinjected. Curve-fitting was performed by minimizing the sum of the least squares for the parameters a and b for a saturation curve of the form I(t) = *a*(1 − e^−*b*t^) [62], where I(t) is the relative fluorescence intensity and t is time. I_max_ and t_1/2_ were determined from *a* and *ln(2)/b* respectively. This equation is also analogous to the equation used in [44] investigating flow of PI into cells which was derived from Fick’s law.

### Optoinjection of Dextrans

Fluorescein-conjugated dextrans of sizes 3–70 kDa (Sigma-Aldrich Co., USA) were added to make a working concentration of 10 µM. For intact cells, dextrans were added prior to incubation to help draw the dextran through the cell wall and maximize dextran concentration in the apoplast [42]. Enzymatic digestion of the cell walls of intact cells to make protoplasts was performed according to [63] and experiments were performed within 24 hours. Dextrans were added prior to the experiment, no incubation was required.

Single cross-sectional images of porated cells were taken before and 3 minutes after irradiation using the confocal system described in the previous section. The imaging of a bubble upon laser irradiation was used as a marker for successful optoinjection. Normalized cellular fluorescence relative to the background was measured using ImageJ.

## Supporting Information

Text S1 (DOCX)

## References

[1] Lodish H, Berk A, Zipursky S (2000) The Dynamic Plant Cell Wall. Molecular Cell Biology. New York: W. H. Freeman.

[2] Campbell NA (1996) Biology. Fourth ed. The Benjamin/Cummings Publishing Company.

[3] Noueiry AO, Lucas WJ, Gilbertson RL (1994) Two proteins of a plant DNA virus coordinate nuclear and plasmodesmal transport. Cell 76: 925–932. 8124726 10.1016/0092-8674(94)90366-2

[4] Traas JA, Doonan JH, Rawlins DJ, Shaw PJ, Watts J, et al (1987) An actin network is present in the cytoplasm throughout the cell cycle of carrot cells and associates with the dividing nucleus. The Journal of Cell Biology 105: 387–395. 2440896 10.1083/jcb.105.1.387 PMC2114883

[5] Wymer CL, Shaw PJ, Warn RM, Uoyd CW (1997) Microinjection of fluorescent tubulin into plant cells provides a representative picture of the cortical microtubule array. The Plant Journal 12: 229–234.

[6] Bothwell JHF, Brownlee C, Hetherington AM, Ng CK-Y, Wheeler GL, et al (2006) Biolistic delivery of Ca2+ dyes into plant and algal cells. The Plant Journal 46: 327–335 10.1111/j.1365-313X.2006.02687.x 16623894

[7] Chang M, Chou J, Lee H (2005) Cellular Internalization of Fluorescent Proteins via Arginine-rich Intracellular Delivery Peptide in Plant Cells. Plant Cell Physiology 46: 482–488 10.1093/pcp/pci046 15695452

[8] Stevenson DJ, Gunn-Moore FJ, Campbell P, Dholakia K (2010) Single cell optical transfection. Journal of the Royal Society Interface 7: 863–871 10.1098/rsif.2009.0463 PMC2871806 20064901

[9] Antkowiak M, Torres-Mapa ML, Stevenson DJ, Dholakia K, Gunn-Moore FJ (2013) Femtosecond optical transfection of individual mammalian cells. Nature Protocols 8: 1216–1233. 23722260 10.1038/nprot.2013.071

[10] Weber G, Zellbiologie M, Ladenburg D- (1988) Microperforation of Plant Tissue with a UV Laser Microbeam and Injection of DNA into Cells. Naturwissenschaften 36: 1–2.

[11] Badr YA, Kereim MA, Yehia MA, Fouad OO, Bahieldin A (2005) Production of fertile transgenic wheat plants by laser micropuncture. Photochemical & Photobiological Sciences 4: 803–807 10.1039/b503658e 16189555

[12] Guo Y, Liang H, Berns MW (1995) Laser-mediated gene transfer in rice. Physiologia Plantarum 93: 19–24.

[13] Kajiyama S, Shoji T, Okuda S, Izumi Y, Fukusaki E, et al (2006) A novel microsurgery method for intact plant tissue at the single cell level using ArF excimer laser microprojection. Biotechnology and Bioengineering 93: 325–331 10.1002/bit.20709 16193516

[14] Kajiyama S, Joseph B, Inoue F, Shimamura M, Fukusaki E, et al (2008) Transient gene expression in guard cell chloroplasts of tobacco using ArF excimer laser microablation. Journal of Bioscience and Bioengineering 106: 194–198 10.1263/jbb.106.194 18804064

[15] Weber G, Monajembashi S, Wolfrum J, Greulich K-O (1990) Genetic changes induced in higher plant cells by a laser microbeam. Physiologia Plantarum 79: 190–193 10.1034/j.1399-3054.1990.790128.x

[16] Vogel A, Noack J, Hüttman G, Paltauf G (2005) Mechanisms of femtosecond laser nanosurgery of cells and tissues. Applied Physics B 81: 1015–1047 10.1007/s00340-005-2036-6

[17] Awazu K, Kinpara T, Tamiya E (2002) IR-FEL-induced green fluorescence protein (GFP) gene transfer into plant cell. Nuclear Instruments and Methods in Physics Research 483: 571–575.

[18] Tirlapur UK, König K (2002) Targeted transfection by femtosecond laser. Nature 418: 290–291 10.1038/418290a 12124612

[19] Barrett LE, Sul J, Takano H, Bockstaele EJ Van, Haydon PG, et al (2006) Region-directed phototransfection reveals the functional significance of a dendritically synthesized transcription factor. Nature Methods 3: 455–460 10.1038/NMETH885 16721379

[20] Lei M, Xu H, Yang H, Yao B (2008) Femtosecond laser-assisted microinjection into living neurons. Journal of Neuroscience Methods 174: 215–218 10.1016/j.jneumeth.2008.07.006 18687359

[21] Mthunzi P, Dholakia K, Gunn-Moore F (2011) Phototransfection of mammalian cells using femtosecond laser pulses: optimization and applicability to stem cell differentiation. Journal of Biomedical Optics 15: 041507 10.1117/1.3430733 20799785

[22] Zeira E (2003) Femtosecond infrared laser–an efficient and safe in vivo gene delivery system for prolonged expression. Molecular Therapy 8: 342–350 10.1016/S1525-0016(03)00184-9 12907157

[23] Tirlapur UK, König K (1999) Technical advance: near-infrared femtosecond laser pulses as a novel non-invasive means for dye-permeation and 3D imaging of localised dye-coupling in the Arabidopsis root meristem. The Plant Journal 20: 363–370. 10571897 10.1046/j.1365-313x.1999.t01-1-00603.x

[24] LeBlanc ML, Merritt TR, McMillan J, Westwood JH, Khodaparast GA (2013) Optoperforation of single, intact Arabidopsis cells for uptake of extracellular dye-conjugated dextran. Optics Express 21: 14662 10.1364/OE.21.014662 23787655

[25] Nagata T, Nemoto Y, Hasezawa S (1992) Tobacco BY-2 Cell Line as the “HeLa” Cell in the Cell Biology of Higher Plants. International Review of Cytology 132: 1–30.

[26] Takebe I (2004) When I Encountered Tobacco BY-2 Cells! Biotechnology in Agriculture and Forestry. 53: 1–6.

[27] Geelen DN V, Inze DG (2001) A Bright Future for the Bright Yellow-2 Cell Culture. Plant Physiology 127: 1375–1379 10.1104/pp.010708.Plant 11743076 PMC1540165

[28] Tsampoula X, Garcés-Chávez V, Comrie M, Stevenson DJ, Agate B, et al (2007) Femtosecond cellular transfection using a nondiffracting light beam. Applied Physics Letters 91: 053902 10.1063/1.2766835

[29] Durnin J (1987) Exact solutions for nondiffracting beams. I. The scalar theory. Journal of the Optical Society of America A 4: 651–654.

[30] Brown CTA, Stevenson DJ, Tsampoula X, McDougall C, Lagatsky AA, et al (2008) Enhanced operation of femtosecond lasers and applications in cell transfection. Journal of Biophotonics 1: 183–199 10.1002/jbio.200810011 19412968

[31] Indebetouw G (1989) Nondiffracting optical fields: some remarks on their analysis and synthesis. Journal of the Optical Society of America A 6: 150 10.1364/JOSAA.6.000150

[32] Durnin J, Miceli JJ (1987) Diffraction-Free beams. Physical Review Letters 58: 1499–1501. 10034453 10.1103/PhysRevLett.58.1499

[33] Antkowiak M, Torres-Mapa ML, Gunn-Moore F, Dholakia K (2010) Application of dynamic diffractive optics for enhanced femtosecond laser based cell transfection. Journal of Biophotonics 3: 696–705 10.1002/jbio.201000052 20583035

[34] Yao C, Qu X, Zhang Z, Hüttmann G, Rahmanzadeh R (2013) Influence of laser parameters on nanoparticle-induced membrane permeabilization. Journal of Biomedical Optics 14: 054034 10.1117/1.3253320 19895136

[35] Heinemann D, Schomaker M, Kalies S, Schieck M, Carlson R, et al (2013) Gold nanoparticle mediated laser transfection for efficient siRNA mediated gene knock down. PloS ONE 8: e58604 10.1371/journal.pone.0058604 23536802 PMC3594183

[36] Kalies S, Birr T, Heinemann D, Schomaker M, Ripken T, et al (2013) Enhancement of extracellular molecule uptake in plasmonic laser perforation. Journal of Biophotonics 9: 1–9 10.1002/jbio.201200200 23341255

[37] Chakravarty P, Qian W, El-Sayed MA, Prausnitz MR (2010) Delivery of molecules into cells using carbon nanoparticles activated by femtosecond laser pulses. Nature Nanotechnology 5: 607–611 10.1038/nnano.2010.126 PMC2917490 20639882

[38] Wu F-S, Feng T-Y (1999) Delivery of plasmid DNA into intact plant cells by electroporation of plasmolyzed cells. Plant Cell Reports 18: 381–386 10.1007/s002990050590

[39] Ferrando M, Spiess WEL (2001) Cellular response of plant tissue during the osmotic treatment with sucrose, maltose, and trehalose solutions. Journal of Food Engineering 49: 115–127.

[40] Brandizzi F, Irons S, Kearns A, Hawes C (2003) BY-2 cells: culture and transformation for live cell imaging. Current Protocols in Cell Biology Chapter 1: Unit 1.7. doi:10.1002/0471143030.cb0107s19. 18228413

[41] Marchington RF, Arita Y, Tsampoula X, Gunn-Moore FJ, Dholakia K (2010) Optical injection of mammalian cells using a microfluidic platform. Biomedical Optics Express 1: 527–536 10.1364/BOE.1.000527 21258487 PMC3017997

[42] Wu F-S, Cahoon AB (1994) Plasmolysis facilitates the accumulation of protein and DNA into extra-plasmalemma spaces of intact plant cells. Plant Science 104: 201–214.

[43] Stevenson D, Agate B, Tsampoula X, Fischer P, Brown CTA, et al (2006) Femtosecond optical transfection of cells: viability and efficiency. Optics Express 14: 7125–7133. 19529083 10.1364/oe.14.007125

[44] Gu L, Mohanty SK (2011) Targeted microinjection into cells and retina using optoporation. Journal of Biomedical Optics 16: 128003 10.1117/1.3662887 22191939

[45] Rudhall AP, Antkowiak M, Tsampoula X, Mazilu M, Metzger NK, et al (2012) Exploring the ultrashort pulse laser parameter space for membrane permeabilisation in mammalian cells. Scientific Reports 2: 1–5 10.1038/srep00858 PMC3497030 23152947

[46] Torres-Mapa ML, Antkowiak M, Cizmarova H, Ferrier DEK, Dholakia K, et al (2011) Integrated holographic system for all-optical manipulation of developing embryos. Biomedical Optics Express 2: 1564–1575 10.1364/BOE.2.001564 21698019 PMC3114224

[47] Rounds CM, Lubeck E, Hepler PK, Winship LJ (2011) Propidium iodide competes with Ca(2+) to label pectin in pollen tubes and Arabidopsis root hairs. Plant Physiology 157: 175–187 10.1104/pp.111.182196 21768649 PMC3165868

[48] Stiles W (1994) Principles of Plant Physiology. Second ed. New Dehli: Discovery Publishing House.

[49] Granath KA (1958) Solution properties of branched dextrans. Journal of Colloid Science 13: 308–328.

[50] Smit JAM, Van Dijk JAPP, Mennen MG, Daoud M (1992) Polymer size exponents of branched dextrans. Macromolecules 25: 3585–3590 10.1021/ma00039a044

[51] Matsuoka H, Komazaki T, Mukai Y, Shibusawa M, Akane H, et al (2005) High throughput easy microinjection with a single-cell manipulation supporting robot. Journal of Biotechnology 116: 185–194 10.1016/j.jbiotec.2004.10.010 15664082

[52] Cappelleri DJ, Halasz A, Sul J-Y, Kim TK, Eberwine J, et al (2010) Towards A Fully Automated High-Throughput Phototransfection System. JALA 15: 329–341 10.1016/j.jala.2010.03.003 20706617 PMC2917821

[53] Rendall HA, Marchington RF, Praveen BB, Bergmann G, Arita Y, et al (2012) High-throughput optical injection of mammalian cells using a Bessel light beam. Lab on a Chip 12: 4816–4820 10.1039/c2lc40708f 23007197

[54] Cizmár T, Mazilu M, Dholakia K (2010) In situ wavefront correction and its application to micromanipulation. Nature Photonics 4: 388–394 10.1038/NPHOTON.2010.85

[55] Kohli V, Acker JP, Elezzabi AY (2005) Reversible permeabilization using high-intensity femtosecond laser pulses: applications to biopreservation. Biotechnology and Bioengineering 92: 889–899 10.1002/bit.20689 16189821

[56] Beck WA (1929) Determining the Osmotic Value at Incipient Plasmolysis. Transactions of the American Microscopical Society 48: 204–208.

[57] Schinkel H, Jacobs P, Schillberg S, Wehner M (2008) Infrared Picosecond Laser for Perforation of Single Plant Cells. Biotechnology 99: 244–248 10.1002/bit 17614330

[58] Baron-Epel O, Gharyal PK, Schindler M (1988) Pectins as mediators of wall porosity in soybean cells. Planta 175: 389–395 10.1007/BF00396345 24221876

[59] Hunold R, Bronner R, Hahne G (1994) Early events in microprojectile bombardment: cell viability and particle location. The Plant Journal 5: 593–604 10.1046/j.1365-313X.1994.05040593.x

[60] Chen Y, Lagerholm BC, Yang B, Jacobson K (2006) Methods to measure the lateral diffusion of membrane lipids and proteins. Methods 39: 147–153 10.1016/j.ymeth.2006.05.008 16846741

[61] Schneider CA, Rasband WS, Eliceiri KW (2012) NIH Image to ImageJ: 25 years of image analysis. Nature Methods 9: 671–675 10.1038/nmeth.2089 22930834 PMC5554542

[62] Goudriaan J (1979) A Family of Saturation Type Curves, Especially in Relation to Photosynthesis. Annals of Botany 43: 783–785.

[63] Miao Y, Jiang L (2007) Transient expression of fluorescent fusion proteins in protoplasts of suspension cultured cells. Nature protocols 2: 2348–2353 10.1038/nprot.2007.360 17947977

