# Supplementary material for: Femtosecond Optoinjection of Intact Tobacco BY-2 Cells Using a Reconfigurable Photoporation Platform
Source: PLoS One. 2013 Nov 14;8(11):e79235. doi: 10.1371/journal.pone.0079235 (PMC3828288; doi:10.1371/journal.pone.0079235)
Supplement: Text S1 — (DOCX) [file pone.0079235.s001.docx]

**Supplementary information:**

S1. Comparing Gaussian and Bessel beams:

When attempting to compare two different beam types, it is essential that the comparison made is as fair as possible. By matching the Gaussian beam waist to the Bessel beam’s central core radius we ensure that the intensity applied at the image plane is kept the same, we can then consider the depth of focus (DOF) for each beam. For a given beam waist diameter, 2ω_0_, the depth of focus can be defined as twice the Rayleigh length, z_R_ = πω_0_^2^/λ [1]. One Rayleigh length from the beam waist, the area of the beam has expanded to twice that at the beam waist and the intensity has therefore halved because the total power contained within the beam is the same. In the case of an axicon-generated Bessel beam, the propagation length can be tuned independently of the central core radius (r_0_), which is only dependent on beam wavelength and axicon opening angle (α). The on-axis intensity of the Bessel beam varies according to $z^{2}e^{-2z^{2}/z_{\max}^{2}}$[2]. The central core size stays the same but the total power carried in the core changes as the number of rings increases along the direction of propagation. The axial propagation length is usually defined as the point at which the intensity drops to 32.27 % of it’s maximum [1] and can be calculated using z_max_ = ω_0_/(n-1)α [3]. Given the definition of the DOF for the Gaussian beam, we can define the Bessel beam DOF to be the point at which the intensity drops to 50 % of the maximum instead which is determined experimentally.

**Supplementary References:**

1. McGloin D, Dholakia K (2005) Bessel beams: Diffraction in a new light. Contemporary Physics 46: 15–28. doi:10.1080/0010751042000275259.

2. Arlt J, Dholakia K, Soneson J, Wright E (2001) Optical dipole traps and atomic waveguides based on Bessel light beams. Physical Review A 63: 063602. doi:10.1103/PhysRevA.63.063602.

3. Antkowiak M, Torres-Mapa ML, Stevenson DJ, Dholakia K, Gunn-Moore FJ (2013) Femtosecond optical transfection of individual mammalian cells. Nature Protocols 8: 1216–1233.
